# Connectedness to Nature and Public (Skin) Health Perspectives: Results of a Representative, Population-Based Survey among Austrian Residents

**DOI:** 10.3390/ijerph110101176

**Published:** 2014-01-20

**Authors:** Daniela Haluza, Stana Simic, Jan Höltge, Renate Cervinka, Hanns Moshammer

**Affiliations:** 1 Institute of Environmental Health, Center for Public Health, Medical University of Vienna, Kinderspitalgasse 15, A-1090 Vienna, Austria; E-Mails: janhoeltge@gmx.de (J.H.); renate.cervinka@meduniwien.ac.at (R.C.); hanns.moshammer@meduniwien.ac.at (H.M.); 2 Institute of Meteorology, University of Natural Resources and Life Sciences, Vienna, Peter-Jordan-Straße 82, A-1190 Vienna, Austria; E-Mail: stana.simic@boku.ac.at

**Keywords:** melanoma, skin cancer prevention, gender differences, connectedness to nature, questionnaire survey, Public Health

## Abstract

Connectedness to nature (CN) influences motivation to have contact with outdoor natural environments. Spending leisure time in natural environments is beneficial for human health and well-being. Besides these positive effects, health risks of open-air activities are mainly related to unprotected sun light exposure-associated acute and chronic skin hazards. Thus, we conducted a cross-sectional, representative telephone survey among Austrian residents to study the association of perceived CN level with sun-exposure knowledge, tanning habits, and sun protective behaviour. In total, 1,500 study subjects (50.5% females) participated in this questionnaire survey. Although knowledge about tanning and motives to tan were similar among genders, females performed more photoprotective measures and were more connected to nature (all *p* < 0.001) compared to males. Older age and outdoor sport were significant gender-independent predictor variables influencing perceived CN level. Additionally, level of education was relevant in male CN, whereas non-smoking and higher knowledge were predictive of female CN. This survey provides so far unreported empirical data on the relationship between nature connectedness and skin health-relevant recreational habits of Austrian residents. The findings suggest to integrate hitherto neglected gender-specific Public (Skin) Health promotion when counselling on the manifold health advantages of outdoor activities.

## 1. Introduction

Contact with nature and its inherent biodiversity is vital for human beings on the level of all three health dimensions—physical, mental and social well-being and health. Advantageous health effects of spending leisure time outdoors have been constantly reported by various scientific domains. Environmental psychology-based research assessed positive effects of natural surroundings on restoration by means of measuring physiological (e.g., cortisol levels) and psychological (e.g., mood) parameters [1]. Moreover, several investigations reported positive associations between amount of natural environments and health benefits [2,3]. On the other hand, enhancement of physical activity through natural environments and subsequent benefits for fitness and body weight was scope of Public Health-related research. Therein, authors concluded that compared to indoor exercise, outdoor activities have greater positive effects on physical and mental well-being [4,5,6].

Besides environmental hazards such as vector-borne diseases, noise and ambient air pollutants including particulate matter and pollen, disadvantages of open-air activities are mostly associated with harmful health effects related to ultraviolet (UV) light [7,8,9]. Intermittent sun exposure patterns as experienced in outdoor abidance for recreational purposes are a well-known risk factors for the development of melanoma, the deadliest form of skin cancer [10]. Increasing melanoma incidence and mortality rates are an important global health issue with vast economic burden [11,12,13]. Furthermore, solar radiation causes deterioration of physical attractiveness by means of skin aging as well as development of nevi and pigmentary abnormalities with limited clinical significance [14]. Other related adverse outcomes comprise various non-dermatological effects such as the ophthalmologic disorders cataract, pterygium, and maculopathy [15].

Effective use of photoprotective measures including consistently applying proper amounts of sunscreen and wearing sunglasses could prevent health-threatening outcomes of lifestyle-associated sunlight exposure [16]. Also, a shift of melanoma primary body sites from head and neck to the chest in recent years points towards modifications in intentional sun exposure driven by age and gender-related fashion trends and tanning behaviour changes [17]. Thus, getting deeper insight into common health beliefs and motives affecting the success of health promotion campaigns is relevant for decision makers, medical professionals, consumers, and patients. In this context, in recently published papers, we have introduced the wording Public (Skin) Health as an umbrella term for respective Public Health efforts focusing on potential risks of solar radiation exposure [18,19].

In recent decades, the construct of connectedness to nature (CN), which is very similar to nature relatedness, has been increasingly used to assess personal characteristics that influence emotional bonds with outdoor natural environments and pro-environmental habits [20]. Moreover, nature connectedness seems to be a character trait that is relatively stable over time, differs among individuals and influences behavior [21,22]. In accordance with this conceptual approach, people with higher CN levels show more motivation to seek contact to natural spaces than people with low CN [23,24]. Thus, from a Public (Skin) Health perspective and in synopsis with epidemiological knowledge on sunlight-associated skin diseases, CN could be seen as risky health behaviour that stimulates a lifestyle with frequent outdoor leisure time activities.

So far, empirical data on the connection between CN and outdoor sun exposure-related habits are missing. Thus, aim of the present population-based telephone survey was to analyze how perceived CN interacts with sun exposure knowledge, motives to tan, and sun protective behaviours of a cross section of Austrian residents. Additionally, data analysis focussed on gender specificity to provide practical implications for developing target group appropriate strategies for national Public (Skin) Health promotion.

## 2. Methods

### 2.1. Data Collection

The present study was part of the transdisciplinary research project “UVSkinRisk”, approved by the local ethical committee of the Medical University Vienna, Austria (EK662_2010) and conducted in accordance with the principles of the International Declaration of Helsinki [25].

We contracted the independent ISO-certified company Triconsult (Vienna, Austria) to realize a nationwide telephone survey. Data were collected from 3 to 17 August 2011 among Austrian residents aged between 18 and 74 years. To acquire a gender-balanced sample, we did not include the age group 75+ in our analysis due to a biased gender distribution. A random sample of 20,000 telephone numbers representative for the Austrian population by means of age and place of residence was draw from the official national telephone directory list. Until the predisposed number of 1,500 completed interviews was reached, about 11,100 individuals refused study contribution due to unknown reasons. Participation was voluntarily and anonymous. Verbal consent was obtained before start of the interview which was completed in about 10 to 12 min. Survey questionnaire design and software assistance prevented item non-response and missing data [26]. Participants reported on socio-demographic characteristics (age, gender as well as highest education categorized into the (school) levels primary, secondary, and tertiary (*i.e.*, high school/university) and classified their pigmentary phototype (skin types I–VI according to the Fitzpatrick Scale) [27]. Further, study subjects provided information on sunbed use, outdoor sunbathing, predominantly used sun protection factor (SPF) value, occurrence of sunburns (in the last year and in childhood), current tobacco smoking habits (categorized into never smokers, ex-smokers, and smokers), and vigorous outdoor sport activities.

To measure participants baseline knowledge of important skin health risks, we used achievement in a test of five true-false questions that basically tested knowledge related to UV light exposure, skin cancer, and sun protective behaviour, adapted from the literature [18,28,29]. Numbers of correct responses were summed to generate the covariate “knowledge score” with a highest achievable score of five correct answers.

Further, we assessed frequency of eight recommended sun protective habits (basically: “For sun protection I use sunscreen (min. SPF 15)/reapply sunscreen during the day/reapply sunscreen after swimming/avoid midday sun/seek shade/wear a hat/wear protective garments/wear sunglasses.”) using a 5-point Likert scale scoring from “always” (=1) to “never” (=5) adapted from the literature [18,30]. To generate a scale on sun protection, we comprising these eight items showing an acceptable internal consistency (Cronbach’s alpha: 0.73). For building the covariate “sun protection”, we summed the set of scores and calculated mean of these eight items.

Respondents were requested to rate their degree of agreement with eight statements related to perceived motives to tan (basically: “A tanned skin is desirable because it enhances sex appeal/enhances attractiveness/enhances self-confidence/enhances fitness/enhances body shape/reduces paleness/reduces acne/reduces stretch marks.”) using a 5-point Likert scale scoring from “strongly agree” (=1) to “strongly disagree” (=5). For building a scale, we combined these eight items, showing an acceptable internal consistency (Cronbach’s alpha: 0.64). In order to generate the covariate “motives to tan”, we summed the set of scores and calculated the mean of these eight items.

In our previously published validation study among an Austria study sample, we showed a high correlation between the scale and the single item for CN assessment [24]. Accordingly, to evaluate personal disposition relevant for environment-related human health behavior in the current survey, we solely employed the single-item version to operationalize nature connectedness, using the eleven-step rating question “How would you rate your connectedness to nature?” ranging from “none” (=0) to “very high” (=10).

### 2.2. Statistical Data Analysis

The collected data were statistically processed using EXCEL database (Microsoft, Seattle, WA, USA) and SPSS Version 17.0 (SPSS Inc., Chicago, IL, USA). For all statistical analyses, two-sided level of significance was set at *p* = 0.05. Data were expressed as proportions, means, and standard deviation (SD) values where appropriate. Also, we used median split to obtain dichotomized subgroups of study subjects with more or less pronounced personal attributes, according to similar evaluations [31,32]. Thus, we separated the knowledge score, the scales (sun protection and motives to tan), and the single item question evaluating CN at cut point of ≤ or > than the median (low/high, respectively). Pearson Chi² tests and Mann Whitney U tests were used to analyze group differences for gender (male/female) and CN (low/high).

Further, we studied the influence of predictor variables comprising socio-demographic characteristics and skin health-related aspects on CN (low/high). Using logistic regression analysis, we assessed homogeneity of demographic factors including age group (in years, younger ages *vs.* 60+), educational level (non-tertiary *vs.* tertiary), skin type (fair *vs.* moderate-dark), as well as smoking habits (non-smoker *vs.* smoker), SPF value (non *vs.* higher values), sport activities, sunbed use, sunburns (all: yes *vs.* no), sunbathing (never *vs.* more often), as well as knowledge score, sun protection and motives to tan (all: low *vs.* high) between study subject with low and high CN, respectively. To control for confounding, we calculated both crude (simple regression model) and adjusted (multiple regression model) odds ratios (OR) and 95% confidence intervals (CI) for both genders. As suggested by Koster *et al.*, we only considered factors with a statistically significant difference in distribution of adjusted models as possible explanations [33].

## 3. Results

In total, 1,500 Austrian study subjects (18–74 years, mean 44.7 years, SD 15.4) participated in this cross-sectional survey. As we intended to acquire a gender-balanced sample, merely slightly more females than males (50.5% *vs.* 49.5%, respectively) completed the questionnaire-based telephone interview.

As depicted in part I of Table 1, 33.7% of respondents reported that they did not sunbathe in 2010. We found significant gender differences as compared to males more females (*p* = 0.02) denied sunbathing activities. Notably and despite these differences, male and female participants did not differ significantly regarding self-reported amount of sunburns both in the last year (*p* = 0.17) and in childhood (*p* = 0.37). Nevertheless, 31% of participants (33% of males and 28% of females, respectively) reported at least one case of sunburn in the last year. Moreover, 1115 subjects (74%, 76% of males and about 72% of female, respectively) experienced sunburns in childhood. For participants who self-reported habitual outdoor tanning activities, we characterized frequency and duration of this behavior (Table 1, part II). Although we did not reveal gender differences regarding frequency (*p* = 0.108), compared to females, males were more likely to spend more time in the sun in general (*p* = 0.024) and during peak sun hours (from 11 am until 4 pm, *p* = 0.029).

Further, Table 2 provides an overview of overall and gender-specific distributions of responses to the CN-single item. Corresponding to median of respective item responses, factors were dichotomized (low/high) to generate subgroups (CN: low: 0–7, high: 8–10; none = 0, very high = 10; knowledge score: low: 0–4, high: 4–7; weak knowledge = 0, full knowledge = 7; motives to tan: low: 0–4, high: 4–5; fully agree = 1, fully disagree = 5; and sun protection: low: 0–3, high: 3–5; very frequently = 1, never = 5). As presented in Table 3, males and females did not differ regarding achievement in the knowledge test and amount of agreement with motives to tan. In contrast, we revealed significant gender differences (*p* < 0.0001) in amount of performed photoprotective behaviours and overall nature connectedness. More specifically, 63.2% of male study subjects compared to 73.4% of female participants reported high CN.

As a further step, we compared low and high CN specifications in regard of sample characteristics (Table 4). Whereas reported amount of motives to tan and performed sun protective behaviours did not significantly differ among CN subgroups, we found differences in knowledge score (*p* = 0.007), occurrence of sunburn 2010 (*p* = 0.003), and outdoor sport activity (*p* < 0.0001). Additionally, participants with distinct smoking habits significantly differed in their CN (*p* < 0.0001), as 20% of smokers, 21% of ex-smokers, and 60% of non-smokers reported to be highly connected to nature.

Next, we investigated gender-specific possible predictor variables for CN subgroups (Table 5). As only 0.3% of participants were assigned to skin type VI, we consolidated skin types V and VI for multiple regression analysis. In our adjusted regression models (male/female model, respectively: log-likelihood = 916.973/803.490, Cox and Snell *R*² = 0.077/0.095, chi² = 59.261/75.392, both models: *df* = 4, *p* < 0.0001), increasing age (analyzed by age groups), and sport activities (males: OR = 1.42, 95%CI = 1.01–1.99; females: OR = 2.05, 95%CI = 1.43–2.93) significantly influenced perceived level of CN gender-independently. 

**Table 1:** Gender differences of (part I) reported sunburns and sunbaths as well as (part II) frequency and duration of outdoor tanning activities.

Factors	Males		Females		Total	
	*n*	%	*n*	%	*n*	%
**Part I.** Total	742	100	758	100	1,500	100
Sunburns 2010; n/year^ a^						
Never	495	66.7	544	71.8	1,039	69.3
1–2	216	29.1	183	24.1	399	26.6
3–5	28	3.8	29	3.8	57	3.8
6–10	3	0.4	2	0.3	5	0.3
*p*	0.170					
Sunburns in childhood; n^ b^						
Never	176	23.7	209	27.6	385	25.7
1*–*2	213	28.7	213	28.1	426	28.4
3*–*5	201	27.1	188	24.8	389	25.9
6*–*10	152	20.5	148	19.5	300	20.0
p	.370					
Sunbaths 2010 ^c^						
Yes	513	69.1	481	63.5	994	66.3
No	229	30.9	277	36.5	506	33.7
p	0.020 *					
**Part II.** Total	513	100	481	100	994	100
Frequency; days ^d^						
1*–*5	157	30.6	130	27.0	287	28.9
6*–*15	148	28.8	154	32.0	302	30.4
16*–*30	128	25.0	126	26.2	254	25.6
>30	80	15.6	71	14.8	151	15.2
p	0.108					
Duration; hours ^e^						
<0.5	95	18.5	109	22.7	204	20.5
0.5*–*1	115	22.4	112	23.3	227	22.8
1–3	172	33.5	165	34.3	337	33.9
>3	131	25.5	95	19.8	226	22.7
p	0.024 *					
During midday hours; hours ^f^						
Never	71	13.8	90	18.7	161	16.2
<1	155	30.2	140	29.1	295	29.7
1*–*2	140	27.3	140	29.1	280	28.2
2*–*4	90	17.5	70	14.6	160	16.1
>4	57	11.1	41	8.5	98	9.9
p	0.029 *					

^a^ In the past year, how many times did you receive a sunburn? ^b^ In your childhood, how many times did you receive a sunburn? ^c^ In the past year, did you usually sunbath outdoors on a sunny day? ^d^ In the past year, how many days did you sunbath outdoors? ^e^ In the past year, how long did you sunbath outdoors on a sunny day? ^f^ In the past year, how often did you sunbath outdoors during midday hours? * *p* < 0.05 from Chi² tests (males *vs.* females).

**Table 2:** Gender-specific distribution of responses to the question “How would you rate your connectedness to nature?” (CN) ranging from “none” (=0) to “very high” (=10).

CN	Males		Females		Total	
	*n*	%	*n*	%	*n*	%
Total	742	100	758	100	1,500	100
0 (none)	2	0.3	2	0.3	4	0.3
1	2	0.3	2	0.3	4	0.3
2	7	0.9	4	0.5	11	0.7
3	8	1.1	1	0.1	9	0.6
4	14	1.9	8	1.1	22	1.5
5	80	10.8	61	8.0	141	9.4
6	41	5.5	38	5.0	79	5.3
7	119	16.0	86	11.3	205	13.7
8	176	23.7	197	26.0	373	24.9
9	84	11.3	88	11.6	172	11.5
10 (very high)	209	28.2	271	35.8	480	32.0

**Table 3:** Gender differences regarding factor specifications of connectedness to nature (low/high CN), knowledge score, motives to tan, and sun protection.

Factors	Mean ^1^	SD	Median	Low; *n* (%) ^2^	High; *n* (%)	Total; *n* (%)
CN ^a^						
Males	7.86	1.95	8	273 (36.8)	469 (63.2)	742 (100)
Females	8.26	1.79	8	202 (26.6)	556 (73.4)	758 (100)
*p*	0.0001 *			0.0001 *		
Total	8.06	1.88	8	475 (31.7)	1,025 (68.3)	1,500 (100)
Knowledge score ^b^						
Males	4.28	1.2	4	158 (21.3)	584 (78.7)	742 (100)
Females	4.32	1.1	4	161 (21.2)	597 (78.8)	758 (100)
*p*	0.550			0.980		
Total	4.3	1.14	4	319 (21.3)	1,181 (78.7)	1,500 (100)
Motives to tan ^c^						
Males	3.86	0.84	4	404 (54.4)	338 (45.6)	742 (100)
Females	3.78	0.91	3.9	434 (57.3)	324 (42.7)	758 (100)
*p*	0.082			0.273		
Total	3.82	0.88	4	838 (55.9)	662 (44.1)	1,500 (100)
Sun protection ^d^						
Males	3.1	0.82	3.1	364 (49.1)	378 (50.9)	742 (100)
Females	2.86	0.795	2.9	464 (61.2)	294 (38.8)	758 (100)
*p*	0.0001 *			0.0001 *		
Total	2.98	0.82	3.0	828 (55.2)	672 (44.8)	1,500 (100)

^a^ none=0, very high =10; ^b^ weak knowledge=0, full knowledge=7; ^c^ fully agree=1, fully disagree=5; ^d^ very frequently=1, never=5; * *p* ≤ 0.001; ^1^ Mann Whitney U tests (males *vs.* females); ^2^ Chi² tests (low *vs.* high).

**Table 4:** Differences in sample characteristics regarding specifications of connectedness to nature (CN, low/high).

Factors	Low CN		High CN		Total	
	*n*	%	*n*	%	*n*	%
**Total**	475	100	1025	100	1500	100
Smoking ^a^						
Non-smoker	231	48.6	610	59.5	841	56.1
Ex-smoker	100	21.1	213	20.8	313	20.9
Smoker	144	30.3	202	19.7	346	23.1
*p*	0.0001 *					
Sport activity ^b^						
Yes	264	55.6	678	66.1	942	62.8
No	211	44.4	347	33.9	558	37.2
*p*	0.0001 **					
Sunburn 2010 ^c^						
Yes	171	36.0	290	28.3	461	30.7
No	304	64.0	735	71.7	1039	69.3
*p*	0.003 *					
Knowledge score						
low	121	25.5	198	19.3	319	21.3
high	354	74.5	827	80.7	1181	78.7
*p*	0.007 *					
Motives to tan						
low	244	51.4	488	47.6	732	48.8
high	231	48.6	537	52.4	768	51.2
*p*	0.183					
Sun protection						
low	214	45.1	508	49.6	722	48.1
high	261	54.9	517	50.4	778	51.9
*p*	0.104					

^a^ Do you smoke or have you ever smoked cigarettes? ^b^ Do you usually do outdoor sports? ^c^ In the past year, did you receive a sunburn? * *p* < 0.05 and ** *p* ≤ 0.001 from Chi² tests (low *vs.* high).

**Table 5:** Logistic regression analysis of factors affecting CN after median split (low CN: 0–7, high CN: 8–10), stratified by gender.

Factors		Males OR (CI 95%)					Females OR (CI 95%)			
	*n*	Adjusted ^1^		Crude		*n*	Adjusted ^2^		Crude	
Total	742					758				
Age; years			*		**			*		**
18–29	160	1 = Ref		1 = Ref		145	1 = Ref		1 = Ref	
30–39	143	2.1 (1.3–3.5)	*	2.3 (1.5–3.7)	**	135	1.5 (0.9–2.6)		1.7 (1.1–2.9)	*
40–49	173	2.4 (1.5–3.8)	**	2.6 (1.6–4.0)	**	167	2.3 (1.3–4.0)	*	2.7 (1.6–4.4)	**
50–59	126	2.6 (1.5–4.5)	**	2.7 (1.6–4.4)	**	134	2.0 (1.–3.6)	*	2.6 (1.5–4.3)	**
60–74	140	1.8 (1.0–3.0)	*	1.8 (1.1–2.8)	*	177	1.8 (1.0–3.3)		2.5 (1.5–4.1)	**
Educational level			*		*					*
Primary	168	1.5 (1.0–2.4)	*	1.4 (1.0–2.1)	*	189	1.3 (0.8–2.2)		1.7 (1.1–2.7)	*
Secondary	320	1.5 (1.1–2.2)	*	1.5 (1.1–2.1)		386	1.4 (0.9–2.1)		1.5 (1.1–2.3)	*
Tertiary	254	1 = Ref		1 = Ref		183	1 = Ref		1 = Ref	
Skin type										
I	25	1 = Ref		1 = Ref		54	1 = Ref		1 = Ref	
II	218	1.2 (0.5–2.9)		1.1 (0.5–2.6)		223	1.5 (0.8–3.0)		1.2 (0.6–2.3)	
III	317	0.9 (0.3–2.1)		0.8 (0.3–1.8)		340	1.8 (0.9–3.5)		1.6 (0.8–2.9)	
IV	108	1.4 (0.5–3.6)		1.2 (0.5–2.9)		96	3.1 (1.3–7.2)	*	2.7 (1.3–5.9)	*
V/VI	74	1.5 (0.6–4.3)		1.4 (0.5–3.7)		45	2.3 (0.9–6.3)		1.9 (0.8–4.7)	
Smoking ^a^										*
Non-smoker	201	1.6 (1.1–2.3)	*	1.7 (1.2–2.4)	*	145	1.6 (1.1–2.6)	*	1.9 (1.3–2.9)	**
Ex-smoker	178	1.4 (1.0–2.2)		1.6 (1.0–2.6)	*	135	1.1 (1.1–1.9)	*	1.4 (0.9–2.3)	
Smoker	363	1 = Ref		1 = Ref		478	1 = Ref		1 = Ref	
SPF value^ b^										
None	157	1 = Ref		1 = Ref		100	1 = Ref		1 = Ref	
1-15	159	1.0 (0.6–1.7)		0.6 (0.4–1.0)	*	177	0.8 (0.4–1.5)		1.3 (0.7–2.4)	
16–25	194	1.2 (0.7–2.0)		0.7 (0.4–1.1)		204	1.0 (0.5–1.9)		0.9 (0.5–1.4)	
26–30	124	1.1 (0.6–1.9)		0.9 (0.5–1.4)		131	1.2 (0.6–2.6)		1.0 (0.6–1.6)	
>30	108	1.2 (0.7–2.2)		0.8 (0.5–1.4)		146	1.1 (0.5–2.2)		1.2 (0.7–2.1)	
Sport activity ^c^			*		*			**		**
Yes	482	1.4 (1.0–2.0)		1.4 (1.0–1.9)		460	2.0 (1.4–2.3)		1.9 (1.4–2.6)	
No	260	1 = Ref		1 = Ref		298	1 = Ref		1 = Ref	
Sunbathing; days ^d^										
Never	229	1 = Ref		1 = Ref		277	1 = Ref		1 = Ref	
<15	386	1.3 (0.8–1.9)		1.1 (0.8–1.6)		407	0.9 (0.6–1.3)		0.8 (0.5–1.1)	
>15	208	1.5 (0.9–2.3)		1.3 (0.9–1.9)		197	0.7 (0.4–1.2)		0.7 (0.5–1.1)	
Sunbed use ^d^					*					*
Yes	64	1 = Ref		1 = Ref		70	1 = Ref		1 = Ref	
No	678	1.5 (0.8–2.6)		1.7 (1.2–2.4)		688	1.4 (0.8–2.5)		1.9 (1.3–2.9)	
Sunburn 2010 ^e^										**
Yes	247	1 = Ref		1 = Ref		214	1 = Ref		1 = Ref	
No	461	1.0 (0.7–1.4)		1.1 (0.8–1.5)		544	1.5 (1.0–2.2)		1.9 (1.3–2.7)	
Knowledge score								*		*
low	158	1 = Ref		1 = Ref		161	1 = Ref		1 = Ref	
high	584	1.2 (0.8–1.7)		1.3 (0.9–1.9)		319	1.6 (1.0–2.3)		1.6 (1.1–2.3)	
Motives to tan										
low	344	1 = Ref		1 = Ref		388	1 = Ref		1 = Ref	
high	398	1.1 (0.8–1.5)		1.1 (0.8–1.6)		370	1.2 (0.8–1.7)		1.4 (1.0–1.9)	
Sun protection					*					
low	305	1.3 (0.9–1.8)		1.4 (1.1–2.0)		417	0.8 (0.5–1.1)		0.9 (0.6–1.2)	
high	437	1 = Ref		1 = Ref		341	1 = Ref		1 = Ref	

^a^ “Do you smoke or have you ever smoked cigarettes?” ^b^ “What is the SPF value of the sunscreen you are usually using?” ^c^ “Do you usually do outdoor sports?” ^d^ “In the past year, how often did you sunbath outdoors?” ^d^ “In general, do you use sunbeds?” ^e^ “In the past year, how many times did you receive a sunburn?” ^1^ log-likelihood = 916.973, Cox and Snell R^²^ = 0 .077, chi² = 59.261, df = 4, *p* < 0.0001; ^2^ log-likelihood = 803.490, Cox and Snell R^²^ = 0.095, chi^²^ = 75.392, df = 4, *p* < 0.0001; * *p* < 0.05, ** *p* ≤ 0.001.

Further, educational level was a relevant factor for CN of male study subjects (primary education: OR = 1.5, 95%CI = 1.0–2.4; secondary education: OR = 1.5, 95%CI = 1.1–2.2), whereas smoking habits (non-smoking: OR = 1.6, 95%CI = 1.1–2.6; ex-smoking: OR = 1.1, 95%CI = 1.1–1.9) and knowledge (OR = 1.6, 95%CI = 1.0–2.3) predicted CN of females.

## 4. Discussion

The present cross-sectional study aimed at providing so far lacking empirical insight into lifestyle habits influencing recreational exposure to outdoor UV radiation. Thus, we conducted telephone interviews among a sample (*n* = 1,500, 50.5% females) representative of the Austrian socio-demographic population in terms of age and place of residence.

In line with previously published research, we herein confirmed evidence for gender differences concerning leisure sun protective and exposure behaviour [34,35,36,37]. In our sample, males compared to females were more likely to spent time in the sun (*p* < 0.05) and reported on using less photoprotective measures such as sunscreen (*p* < 0.0001).

Moreover, overall sun exposure-related knowledge reached quite satisfactory levels among both genders, with as many as 79% of study subjects achieving a high knowledge score. Nevertheless, it seems that this knowledge has not been put into execution: nearly one third of study subjects suffered from sunburn in 2010 and even about three quarters reported childhood sunburns. These are alarmingly high percentages as it is evident that a brief, intense sun exposure pattern resulting in blistering sunburn rather than years of tanning might cause melanoma [38]. However, in addition to behavioural aspects, genetic factors such as a family disposition, multiple nevi, and skin type could influence one individual’s melanoma risk [39]. In addition to early diagnosis of skin lesions, reducing the amount of blistering sunburns are the most important means to challenge worldwide rising melanoma incidence and mortality rates [40]. Our study findings suggest that on the one hand previous Public (Skin) Health campaigns effectively transported educative information to the Austrian population (high knowledge). On the other hand, there is still an urgent need to further increase health risk awareness of unprotected sun exposure, achievable by, e.g., public melanoma prevention campaigns [19].

Nature connectedness was consistently associated with emotional dimensions of experiences in nature environments and associated feelings of mindfulness and eudemonic aspects of well-being [24,41]. As a possible explanation, contact to nature perhaps provokes and enhances physical health which, consecutively, could increase mental well-being or *vice versa*. Thus, we analyzed the relationship between CN level and spending leisure time outdoors for recreational tanning behaviour and sport activities. Common tools to assess emotional bondages with outdoor environments are constantly advanced and refined [22,42]. Established measures suitable for evaluating relatedness to nature include a scale and a single-item version to operationalize CN. Short-form versions of pre-existing CN scales were reliable measurement tools in other related studies [43]. Accordingly, based on a previous study that reported a high correlation between the scale and the single item, we used the latter to assess CN [24]. Advantages of this measure included the possibility to explore the concept of CN fast and easily as well as the unbiased neutral wording.

Generally, 27% of males and 19% of females were tobacco smokers. These findings correlate exactly with previously published smoking rates, indicating high reproducibility of smoking incidence data in Austria [44]. In addition to what this survey adds to skin health-related aspects, we found so far unreported associations of CN with current tobacco consuming habits. In order to potentially reveal new aspects of inducing behavioural modifications, future research could address the question why smokers were less connected to nature (*p* < 0.0001, Table 4). In our population-based sample, 30% of participants with low CN levels smoked, whereas regarding high CN, 20% of study subjects were smokers, 21% ex-, and 60% non-smokers. This finding was unexpected as to date, in contrast to indoor premises smoking in outdoor environments including parks and urban green spaces has not yet been regulated by Austrian law. However, from a Public Health perspective, a legal smoking ban would be favourable regarding smoking cessation encouragement and second-hand smoke exposure reduction [45]. Additionally, a smoking stop could directly improve health-related quality of life [46].

Further, we confirmed recently reported observations regarding the impact of socio-demographic characteristics on CN. We found an overall significant impact of both age (young *vs.* older age) and gender (females *vs.* males) [47]. In our survey, compared to people reporting low CN, more study participants reporting high CN performed outdoor sport activities (*p* < 0.0001). This observation was gender-independent and is in line with existing knowledge on the influence of CN on amount of time spent in natural environments [23,24]. On the contrary, staying outdoors for the purpose of sunbathing as well as indoor tanning were not significant predictive factors for perceived level of CN. Additionally, we did not reveal predictive potential for phenotype, *i.e.*, skin type, and tanning behaviour-related aspects (occurrence of sunburn, SPF value, motives to tan, and sun protection).

It is commonly accepted understanding that green exercise, *i.e.*, physical exercise whilst being exposed to natural environments, has positive effects on well-being. Additionally, Watson *et al.* showed that the lately booming urban and “guerrilla” gardening in Western countries, community as well as private gardening exerted various health-promoting outcomes including cardiovascular and restorative benefits [48]. Accordingly, spending 15 min in outdoor environments increased CN, attention span, and positive feelings through nature’s vast interactions with the human socio-biology [49]. Also, besides primary and secondary preventive measures, green exercise could offer mood-stabilizing and activity-promoting opportunities in rehabilitative settings [50]. In the same verve, researchers showed that respective environmental education programs increased children’s CN [51]. To comply with Public (Skin) Health demands, these programs should also comprise evidence-based educative information on effective photoprotective behaviours to reduce UV radiation-associated skin hazard [18].

Although natural spaces could offer plentiful resources for people’s health promoting activities, there is a need to raise awareness on the potential health risks of staying outdoors, as recommended by Kamioka *et al.* [52]. This suggestion is based on the notion that in contrast to the vast amount of literature identifying advantages outcomes of spending time outdoors, potential skin risks have been neglected in investigations of physico-chemical, but also psycho-mental effects of exposure to green spaces [1]. For example, unprotected extensive outdoor sport activities like marathon running was reported to increase melanoma risk [53]. Further, occupational UV radiation exposure of outdoor workers was also shown to be a causative factor for all types of skin cancer [54]. Nevertheless, in contrast to lifestyle-associated sun exposure, e.g., for tanning purposes, photoprotective measures during work-related outdoor abidance are legally controlled by occupational medicine-related regulations [55].

Despite potential short- and long-term health risks, UV radiation exposure was promoted as a surplus benefit of intentional outdoor activities due to its potential to increase vitamin D blood levels. Respective recommendations are contradicting Public (Skin) Health promoting efforts aimed at reducing sun light exposure and could create confusion and reluctance in consumers and patients. These considerations should be integrated in the debate regarding encouragement of spending time outdoors in natural environments in order to enjoy the aforementioned merits on well-being.

As strength of this study, the current findings add to a growing body of literature on positive effects of nature that low and high levels of perceived CN differed in recreational outdoor activities. In synopsis with our findings on gender differences in regard of health risky behaviour (including solar protection and tobacco smoking), results of this survey contribute additional evidence that the conventional “one-size-fits-all” approach might not be the best way to successful skin health promotion strategies [56].

The findings in this report are subject to several limitations. First, a non-response bias could not be ruled out because participation in the study was voluntary and Austrian citizens that chose to answer the questionnaire may have been more concerned about their skin health or sun protective behaviour. Second, this survey assessed self-reported data introducing social desirability and recall bias. Nevertheless, recall bias regarding UV light exposure was shown to be quite small [57]. Third, dichotomization by means of median splits could have methodological consequences such as loss of information [58]. However, in a wide range of scientific disciplines, median split is a commonly used method for analysis of survey data. As we considered size and distribution of our study sample to allow for dichotomizing variables, we also applied this technique in the present survey. Last, causal relations between CN and sun-related behaviour could not be drawn due to the cross-sectional design of this survey. Nonetheless, contact with nature could create positive effects on well-being and thus could promote intentional activities to increase the time spent outdoors, leading to UV radiation exposure.

## 5. Conclusions

The results of this cross-sectional telephone survey among Austrian residents provide theoretical contributions to the understanding of the relationship between the construct connectedness to nature and skin health-related aspects. Further, a so far unrecognized overlap between environmental psychology and Public (Skin) Health was identified. Thus, we suggest interdisciplinary networks including various expert groups such as landscape architects, environmental psychologists, and medical professionals to further examine benefits and risks of recreational outdoor nature experiences.
